# The relationship between motorcycle riders causing traffic accidents, personality disorders and drug abuse 

**Published:** 2019-08

**Authors:** Hasan Janfada, Mohamad Hadi Farahzadi, Fatemeh Sorayani, Mohamad Hasan Lotfi, Reza Ali Falahzade

**Affiliations:** ^*a*^Shahid Sadoughi University of Yazd, Yazd, Iran.

## Abstract

**Background:**

In this research, the relationship between motorcycle riders causing traffic accidents, personality disorders and drug abuse is studied.

**Methods:**

This was a descriptive cross-sectional study (1397) and a sample of 100 motorcyclists in the accident city of Yazd. The data were collected by referring directly to motorcyclists through a two-part questionnaire, demographic characteristics and MMPI personality test. The samples were randomly selected.

**Results:**

Occupation of people and the use of morphine is related but the occupation is not effective on the rest of the cases. An economic feature is effective on the use of methadone. And the economic attribute is effective on the causes of personality disorders such as self-immolation, hysteria, psychosis and mania. The history of previous accidents with substance abuse has a significant relationship. But the history of previous accidents with personality disorders has no meaningful relationship. In accidents, according to the characteristics of rider years and substance abuse, there is a significant relationship. And only has a meaningful relationship with psychiatric personality disorder, and there is no relationship between accidents based on rider years and personality disorder. In accidents, there is a significant relationship between the use of other vehicles in motorcyclists and substance abuse. However, the use of other vehicles in motorcycle riders and personality disorder is not significant. In accidents, there is a significant relationship between the characteristics of having or not having a license for motorcyclists and the use of morphine. Also, there is a meaningful relationship in accidents based on having or not having a motorcyclist certificate and personality disorder, self-medication, depression and hysteria. There is no meaningful relationship in accidents based on the specifics of police fining in motorcyclists and substance abuse. Also, there is a meaningful relationship in accidents based on police fines in motorcycle riders and personality disorder, depression, anxiety, obsession and mania.

**Conclusions:**

According to what was reported in the findings, substance abuse and personality disorder generally increase the incidence of accidents.

**Keywords:**

Substance abuse, Personality disorder, Motorcycle riders

